# Psychopathological network for early-onset post-stroke depression symptoms

**DOI:** 10.1186/s12888-023-04606-1

**Published:** 2023-02-21

**Authors:** Chensheng Pan, Guo Li, Wenzhe Sun, Jinfeng Miao, Yanyan Wang, Yan Lan, Xiuli Qiu, Xin Zhao, He Wang, Zhou Zhu, Suiqiang Zhu

**Affiliations:** Department of Neurology, Tongji Hospital, Tongji Medical College, Huazhong University of Science and Technology, No 1095 Jiefang Avenue, Qiaokou District, Wuhan, Hubei 430030 China

**Keywords:** Early-onset post-stroke depression symptoms, Psychopathological network, Voxel-based lesion-symptom mapping, Lesion location

## Abstract

**Background:**

Post-stroke depression (PSD) can be conceptualized as a complex network where PSD symptoms (PSDS) interact with each other. The neural mechanism of PSD and interactions among PSDS remain to be elucidated. This study aimed to investigate the neuroanatomical substrates of, as well as the interactions between, individual PSDS to better understand the pathogenesis of early-onset PSD.

**Methods:**

A total of 861 first-ever stroke patients admitted within 7 days poststroke were consecutively recruited from three independent hospitals in China. Sociodemographic, clinical and neuroimaging data were collected upon admission. PSDS assessment with Hamilton Depression Rating Scale was performed at 2 weeks after stroke. Thirteen PSDS were included to develop a psychopathological network in which central symptoms (i.e. symptoms most strongly correlated with other PSDS) were identified. Voxel-based lesion-symptom mapping (VLSM) was performed to uncover the lesion locations associated with overall PSDS severity and severities of individual PSDS, in order to test the hypothesis that strategic lesion locations for central symptoms could significantly contribute to higher overall PSDS severity.

**Results:**

*Depressed mood*, *Psychiatric anxiety* and *Loss of interest in work and activities* were identified as central PSDS at the early stage of stroke in our relatively stable PSDS network. Lesions in bilateral (especially the right) basal ganglia and capsular regions were found significantly associated with higher overall PSDS severity. Most of the above regions were also correlated with higher severities of 3 central PSDS. The other 10 PSDS could not be mapped to any certain brain region.

**Conclusions:**

There are stable interactions among early-onset PSDS with *Depressed mood*, *Psychiatric anxiety* and *Loss of interest* as central symptoms. The strategic lesion locations for central symptoms may indirectly induce other PSDS via the symptom network, resulting in higher overall PSDS severity.

**Trial registration:**

URL: http://www.chictr.org.cn/enIndex.aspx; Unique identifier: ChiCTR-ROC-17013993.

**Supplementary Information:**

The online version contains supplementary material available at 10.1186/s12888-023-04606-1.

## Introduction

Post-stroke depression (PSD) is a common complication of stroke, affecting about 29% of patients at any time within 5 years poststroke [1]. PSD is correlated with reduced quality of life, less treatment utilization, poorer functional outcomes and higher long-term mortality [2]. Vast majority of studies on the biopsychosocial determinants of PSD are based on a dichotomized PSD diagnosis or the sum score of a depression scale [3]. Both Diagnostic and Statistical Manual of Mental Disorders, fifth edition (DSM-5) criteria and sum scores are based on the traditional common cause theory, assuming that depression as an entity causes various symptoms and these symptoms are interchangeable and diagnostically equivalent [4–6]. The depressive symptoms, however, actually interact with each other in complex ways, which has long been common knowledge among clinicians [4–6]. In the recent psychopathological network theory, mental disorders are conceptualized as dynamic and complex networks of symptoms influencing each other by creating causal pathways and feedback loops [4–6]. Symptoms most highly connected with other symptoms in a network (so called central symptoms) are more likely to be responsible for triggering or sustaining the rest of symptoms (peripheral symptoms) and may serve as effective targets for psychosocial interventions [5]. In depression, for example, *depressed mood* is consistently reported as a central node [7, 8] and can induce subsequent *insomnia* and then *fatigue* which in turn leads to deterioration of *depressed mood*. Depression symptom networks have been well-established in neurologically healthy populations [7, 9] and interactions among PSD symptoms (PSDS) may also exist in stroke population and be somewhat dynamic over time [10]. Though the PSDS networks at subacute and chronic stages of stroke have been explored in a recent study [10], the network model of early-onset PSDS is yet to be determined and may present unique features considering the acute cerebral insults without functional reorganization/compensation and a more critical hospitalization setting.

PSDS in stroke patients not only represent the psychological impact of physical disability and cognitive impairment, but are also considered a direct consequence of mood-related neuroanatomical damage [2, 3]. Recent evidence suggested that both early-onset PSDS and right basal ganglia infarction were predictive of future PSDS at chronic stage of stroke [11]. However, the direct association between lesion location and early-onset PSDS remains elusive. There is a growing recognition that some biopsychosocial risk factors for depression may not be equally related to all depressive symptoms [12, 13]. For example, the association between lesion characteristics and PSD was found symptom-specific [14]. Therefore, investigating neural substrates of PSD at the level of individual symptoms or aspects has been recommended in the field of PSD research [15, 16]. Here we applied the voxel-based lesion-symptom mapping (VLSM) technique to investigate the lesion locations associated with various early-onset PSDS. We hypothesized that the neural mechanism and the symptom network model of PSDS could be integrated into a new theory: the relationship between lesion location and overall PSDS severity, if any, might largely be driven by the neuroanatomical correlates of central symptoms.

This study aimed to (1) model early-onset PSDS as a complex network and identify central symptoms; (2) uncover lesion locations associated with overall PSDS severity and severities of individual PSDS using VLSM.

## Methods

### Patients and study design

A multi-center prospective cohort was enrolled from Tongji Hospital, Wuhan Central Hospital and Wuhan First Hospital in Wuhan City, Hubei Province, China, between May 2018 and August 2019. Institutional review boards reviewed and approved all study protocols. Written informed consent was obtained from all participants. The inclusion criteria were: (1) acute stroke confirmed with magnetic resonance imaging (MRI) or computed tomography (CT), with symptom onset to hospital admission < 7 days, (2) age ≥ 18 years old. The exclusion criteria were: (1) brain dysfunction caused by non-vascular causes, (2) history of depression, dementia and other psychiatric disorders, (3) communication problems due to aphasia, severe dysarthria, disturbance of consciousness, (4) unable to complete the follow-up, (5) transient ischemic attack and subarachnoid hemorrhage, (6) other concomitant neurological disorders, such as Parkinson’s disease and epilepsy. For the 1,231 consecutive patients enrolled with the above criteria, baseline information was collected within the first 24 h after admission. We collected age, sex, education years, prior stressful life event, past medical history, stroke type, stroke severity (National Institutes of Health Stroke Scale, NIHSS), cognitive function (Mini-Mental State Examination, MMSE), levels of disability and handicap (Barthel Index, BI, and modified Rankin scale, mRS) and lesion localization. The 17-item Hamilton Depression Rating Scale (HDRS), well validated in PSD screening [17], was applied to assess PSDS at two weeks after stroke onset by two experienced psychiatrists with high interrater reliability, as described elsewhere [18]. Information on antidepressant use was also collected. During data analysis, we further excluded patients with prior stroke history to prevent influence introduced by prior stroke lesions (*n* = 189). Patients were also excluded due to: missing PSDS behavioral data (*n* = 59), neuroimages unavailable or unqualified for lesion delineation (*n* = 122). Finally, a total of 861 patients were included in this study.

### Network analysis

#### Item selection

Among the 17 items in HDRS, item 14 (*Genital symptoms*) was excluded from network analysis due to low reporting rate. Item 17 (*Insight*), showing low variance which might bias the network structure [4], was also excluded. Some items in HDRS may in fact measure the same latent variable (a phenomenon termed topological overlap) and therefore bias the centrality estimates [4]. Items 4–6 (*Initial insomnia, Insomnia during the night, Late insomnia*) have potential topological overlap [4] and were merged into a single item *Insomnia* by the summing approach [9]*.* The resulting 13 items were further checked for topological overlap with the *goldbricker* function (threshold: 0.5) in R package *networktools *[19](version 1.4.0). Since no reduction of nodes was suggested through the *goldbricker* procedure, all 13 PSDS items were included in the network analysis. The scoring range was 0–2 for *Gastrointestinal somatic* and *General somatic* PSDS, 0–6 for *Insomnia* and 0–4 for all other items.

#### Network estimation

In the network model, nodes represent individual PSDS and edges correspond to the interactions between PSDS. The partial correlation between two nodes was estimated while controlling for all other nodes in the network. Regularized Gaussian graphical models were developed based on Spearman correlation using the graphical lasso method and the extended Bayesian Information Criteria. The resulting networks were visualized using the Fruchterman-Reingold algorithm. In the network layout, the thickness of edges reflects the strength of associations among nodes. Green and red edges represent positive and negative associations, respectively. PSDS with stronger and more connections are placed closer to each other and more centrally within the network. All procedures were performed using R packages *bootnet* [20] and *qgraph *[21].

#### Network characterization

Centrality of a node represents the overall strength of connections with other nodes in the network. Centrality indices include betweenness, closeness, strength and expected influence (EI), among which EI is demonstrated to have the greatest reliability and interpretability in recent literature [10, 22]. Therefore, only EI (one-step EI, defined as the weight sum of all edges connected to a node where the sign of edge weight was maintained) was reported in the main text. Definitions and results involving the other three indices were mentioned in the Supplemental Material. Predictability indicates the variance of a node that can be explained by surrounding nodes. Predictability was visualized as a ring-shaped pie chart around a node and was estimated using the R package *mgm.*

#### Network stability and accuracy

The stability of node centrality was estimated by the case-dropping bootstrap approach. The correlation stability coefficient (CS-C) measures the maximum drop proportion to retain correlation of 0.7 with the centralities of the original network in at least 95% of the samples. The preferred threshold for CS-C is above 0.5 [20]. Next, we tested the edge accuracy by the nonparametric bootstrap technique. The 95% confidence intervals of edge weights were calculated with 1,000 bootstraps and narrower confidence intervals indicate higher stability of network structure. Additionally, centrality difference tests and edge weight difference tests were performed to determine whether node centralities or edge weights within a given network differ from each other significantly. All procedures were performed in R package *bootnet* [20].

### Image acquisition and preprocessing

Clinical neuroimages (MRI and/or CT) performed upon admission were collected for all 861 participants. Acquisition parameters were shown in Supplemental Table I. The exact timing of neuroimaging since acute onset was also recorded. The lesions were manually segmented on diffusion weighted imaging for ischemic strokes (*n* = 775) and CT for hemorrhagic strokes (*n* = 86) by an experienced rater (Chensheng Pan) blinded to behavioral data in ITK-SNAP version 3.8.0 (www.itksnap.org). The lesion masks were supervised by another well-trained neurologist (Wenzhe Sun) for agreement. Spatial normalization to Montreal Neurological Institute (MNI 152) template was performed for original MRI/CT images and native lesion masks with Clinical Toolbox [14] of Statistical Parametric Mapping (SPM12, Wellcome Trust Centre for Neuroimaging, London, United Kingdom) running on MATLAB R2021a (The MathWorks, Inc, Natick, MA). Visual inspection of the normalized lesion maps, as well as manual correction if necessary, were performed (Chensheng Pan and Wenzhe Sun). The lesion volume was derived from the normalized lesion map in ITK-SNAP. All lesion maps were overlaid on template to show the lesion distribution of the study sample in MRIcron version 1.0 (Chris Rorden, Columbia, SC; www.nitrc.org/projects/mricron).

### Voxel-based lesion-symptom mapping

VLSM analyses were performed in NiiStat [23] to test the association between lesion location and HDRS sum score, as well as 13 individual PSDS scores. All continuous behavioral measures were de-skewed with the de-skew function of NiiStat to optimize statistical power. Only voxels involving at least five patients were included to maintain statistical power [24]. General linear regression with lesion volume as covariate was used to test the association between lesion status of each voxel and behavioral scores. To control the false positive rate in multiple comparisons, voxel-level family wise error (FWE) correction was performed with 5,000 random permutations [25]. Results were thresholded at *P*(FWE) < 0.05 at voxel level. For identification of significant voxels, the resulting Z statistical map was overlaid onto the Automated Anatomical Labeling 3 (AAL3) [26] and “JHU-WhiteMatter-labels-1 mm” atlases in MRIcron.

## Results

### Sample characteristics

The characteristics of the study sample were shown in Table 1. The behavioral data were listed in Table 2. The prevalence of suprathreshold early-onset PSD (HDRS > 7) was 52.7% (454/861). In terms of individual PSDS, *Insomnia* had the highest prevalence, while *Suicidality* had the lowest. No antidepressant was initiated at the time of behavioral assessment for all participants.

**Table 1:** Sociodemographic and clinical characteristics of the study sample

Variable	N (%) or mean (SD)
N	861
Age, years, mean (SD)	57.98 (11.22)
Sex	
Male, N (%)	658 (76.4)
Female, N (%)	203 (23.6)
Education years, mean (SD)	9.40 (4.15)
Stressful life event, N (%)	45 (5.2)
Vascular risk factors and chronic comorbidities	
Current smoker, N (%)	468 (54.4)
Alcohol consumption, N (%)	424 (49.2)
Diabetes mellitus, N (%)	196 (22.8)
Hypertension, N (%)	497 (57.7)
Hyperlipidemia, N (%)	161 (18.7)
Coronary heart disease, N (%)	59 (6.9)
Stroke type	
Acute ischemic stroke, N (%)	775 (90.0)
Intracerebral hemorrhage, N (%)	86 (10.0)
NIHSS score, mean (SD)	3.85 (3.44)
BI score, mean (SD)	76.40 (27.95)
mRS score, mean (SD)	2.26 (1.42)
MMSE score, mean (SD)	24.64 (4.74)
Lesion localization	
Left hemispheric, N (%)	328 (38.1)
Right hemispheric, N (%)	338 (39.3)
Bihemispheric, N (%)	37 (4.3)
Infratentorial, N (%)	158 (18.3)
Lesion volume, cm^3^, mean (SD)	18.08 (32.69)
Onset to neuroimaging, days, mean (SD)	3.06 (2.16)

*NIHSS* indicates National Institutes of Health Stroke Scale, *BI* Barthel Index, *mRS* modified Rankin scale, *MMSE* Mini-Mental State Examination, *SD* standard deviation

**Table 2:** Behavioral data on early-onset PSDS

Variable	HDRS item	Abbreviation	Prevalence^a^ (%)	Mean (SD)
HDRS sum score	1–17	-	52.7 (HDRS > 7)	9.06 (6.86)
Individual PSDS				
Depressed mood	1	DepMood	55.9	1.16 (1.29)
Feelings of guilt	2	Guilt	51.8	0.60 (0.66)
Suicidality	3	Suic	10.8	0.15 (0.49)
Insomnia	4–6	Insomn	57.1	1.66 (1.88)
Loss of interest in work and activities	7	Workact	52.3	1.27 (1.46)
Retardation	8	Retard	42.5	0.53 (0.71)
Agitation	9	Agit	39.1	0.49 (0.68)
Psychiatric anxiety	10	PsyAnx	56.8	1.07 (1.15)
Somatic anxiety	11	SomAnx	36.0	0.43 (0.63)
Gastrointestinal somatic	12	GISom	25.6	0.28 (0.50)
General somatic	13	GenSom	32.4	0.37 (0.57)
Hypochondriasis	15	Hypochon	30.2	0.46 (0.78)
Weight loss	16	WtLoss	19.9	0.26 (0.55)

*HDRS* indicates Hamilton Depression Rating Scale, *PSDS* post-stroke depression symptoms

^a^ HDRS sum score > 7 indicates presence of suprathreshold depression; an item score ≥ 1 was regarded as presence of the corresponding PSDS

### Network characteristics

**Fig. 1:**
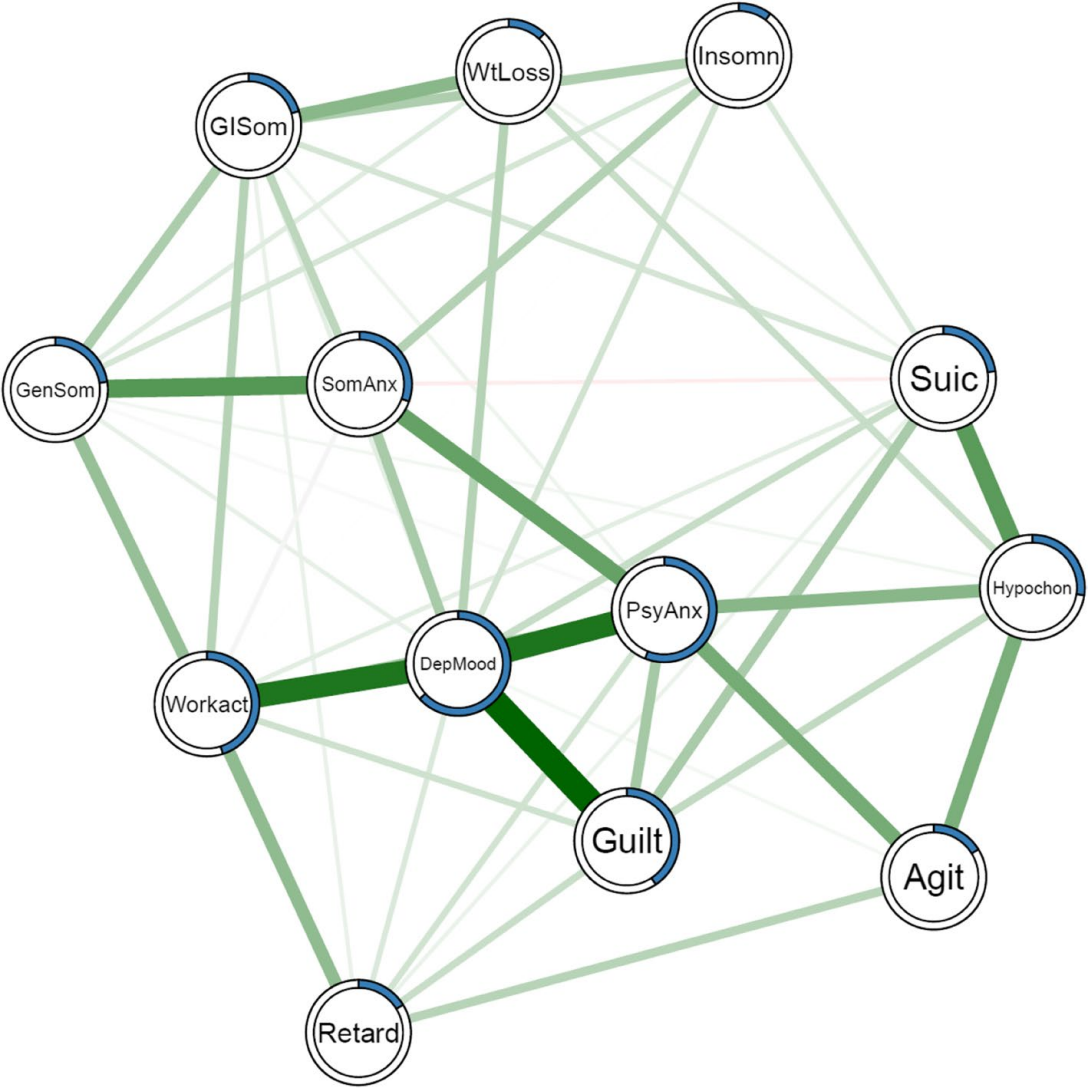
Symptom network at two weeks after stroke. The thickness of edges reflects the strength of associations among nodes. Green and red edges represent positive and negative associations, respectively. PSDS with stronger and more connections are placed closer to each other and more centrally within the network. Pie charts around nodes indicate predictability. DepMood indicates depressed mood; Guilt, guilt feelings; Suic, suicidality; Insomn, insomnia; Workact, loss of interest in work and activities; Retard, retardation; Agit, agitation; PsyAnx, psychiatric anxiety; SomAnx, somatic anxiety; GISom, gastrointestinal somatic symptoms; GenSom, general somatic symptoms; Hypochon, hypochondriasis; WtLoss, weight loss

**Fig. 2:**
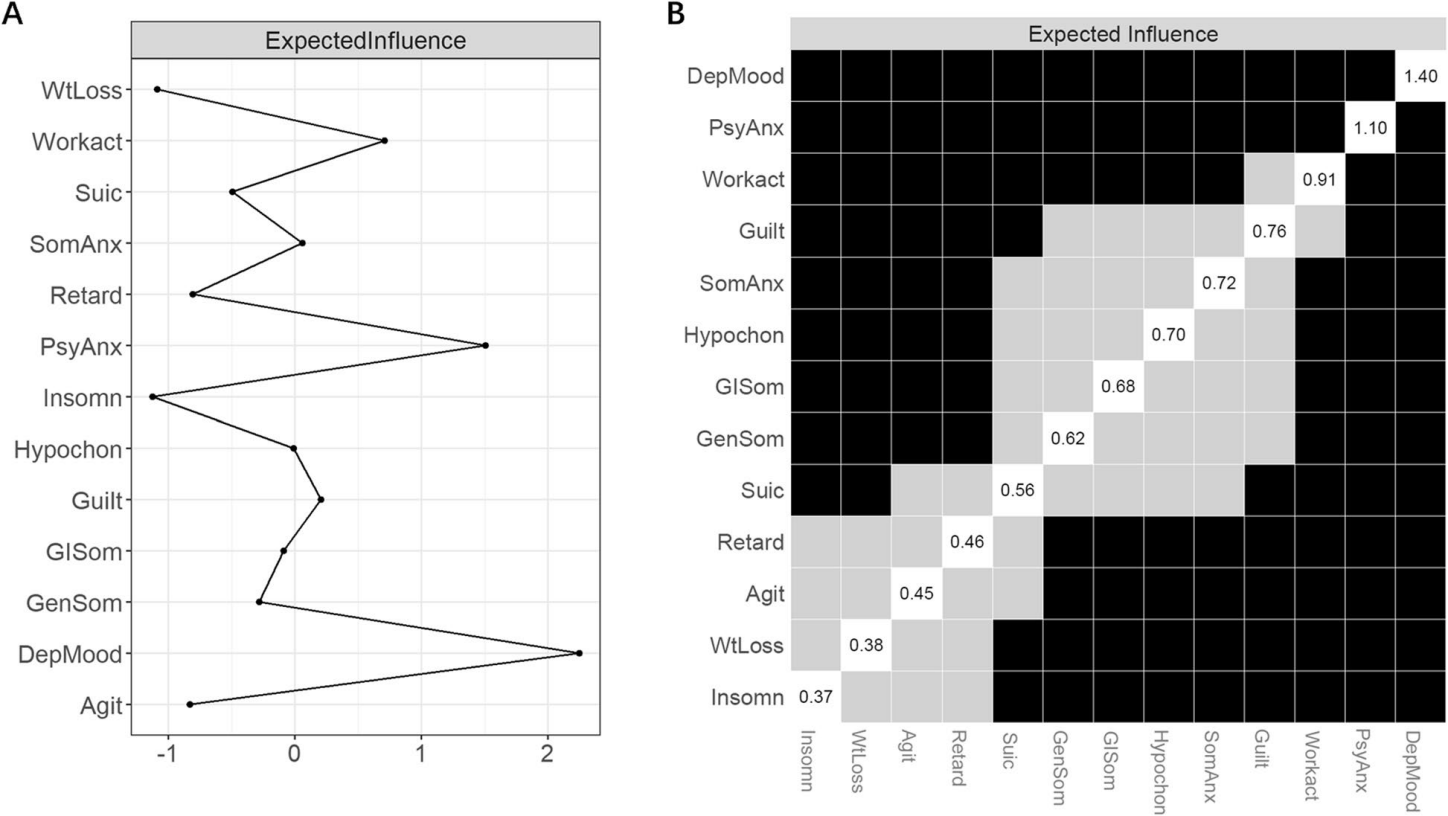
Expected influence (EI) for all nodes. **A**, Z-score standardized EI values; **B**, results of centrality difference tests: black boxes indicate significant differences between two nodes, grey boxes indicate non-significant differences, the number in the white boxes indicate the value of EI. DepMood indicates depressed mood; Guilt, guilt feelings; Suic, suicidality; Insomn, insomnia; Workact, loss of interest in work and activities; Retard, retardation; Agit, agitation; PsyAnx, psychiatric anxiety; SomAnx, somatic anxiety; GISom, gastrointestinal somatic symptoms; GenSom, general somatic symptoms; Hypochon, hypochondriasis; WtLoss, weight loss

The PSDS network was visualized in Fig. 1. Standardized EI values for all nodes were shown in Fig. 2A. *Depressed mood* had the highest centrality, followed by *Psychiatric anxiety* and *Loss of interest in work and activities* which had significantly larger EI values than most other nodes (Fig. 2B). Closeness, betweenness and strength for all nodes were shown in Supplemental Figures I and II. The top three strongest connections were observed among *Depressed mood – Guilt feeling, Depressed mood – Psychiatric anxiety,* and *Depressed mood – Loss of interest in work and activities* (Supplemental Figure III)*.*

### Network stability and accuracy

**Fig. 3:**
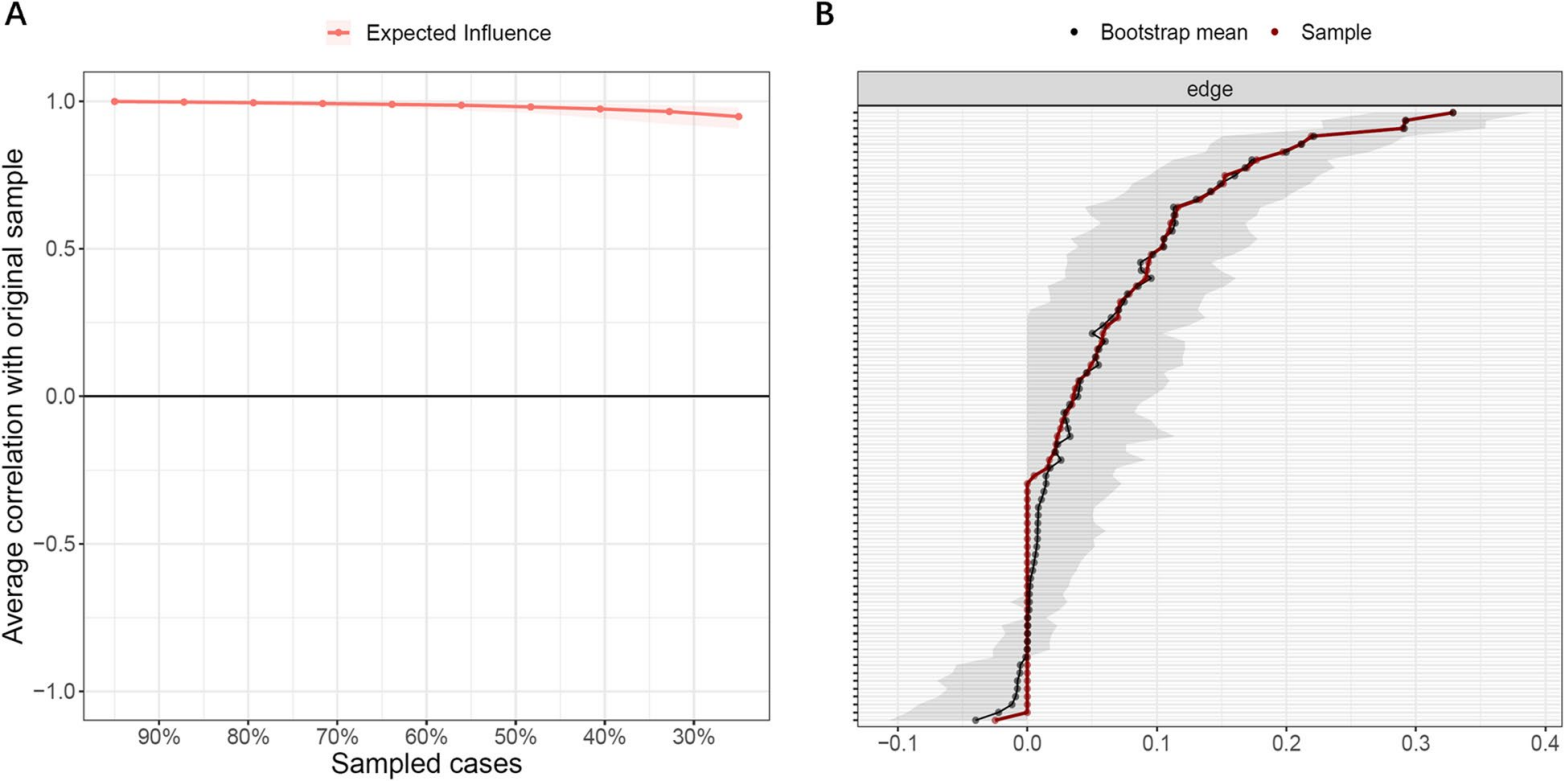
Network stability and accuracy. **A**, Stability of expected influence in 1,000 case-dropping bootstraps. Correlation Stability Coefficient (CS-C) for expected influence: 0.75; **B**, Bootstrap 95% confidence intervals for estimated edge weights of the symptom network. Each horizontal line represents one edge. Edge weights are represented by the red line. The 95% confidence intervals are indicated by the grey area

The EI showed excellent stability (Fig. 3A) with a CS-C of 0.75. Edge weight stability plot (Fig. 3B) suggested relatively high accuracy of edge weights and acceptable stability of the network structure.

### Voxel-based lesion-symptom mapping

**Fig. 4:**
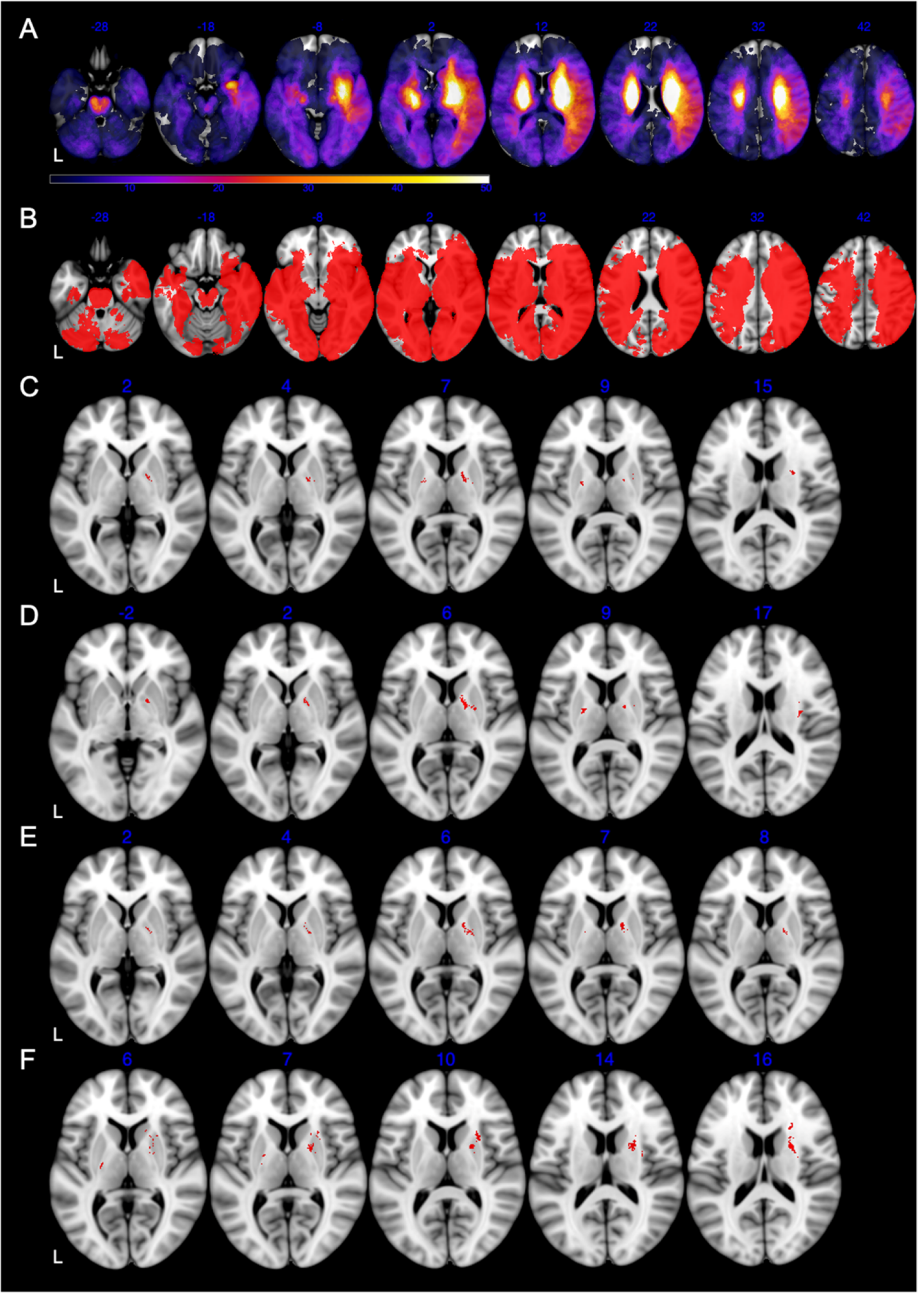
Results of VLSM analyses. **A**, lesion overlap map (*n* = 861), color bar indicates number of participants with lesion at each voxel; **B**, lesion coverage map, only voxels lesioned in at least 5 patients were included in VLSM (red); **C**, significant voxels for higher HDRS sum score (red); **D**-**F**, significant voxels for higher *Depressed mood, Loss of interest, Psychiatric anxiety* scores, respectively (red). Axial coordinates refer to MNI space in mm. L indicates left

The lesion overlap map was shown in Fig. 4A to characterize the study sample. After excluding rarely involved voxels, 54.7% (998,780/1,827,243) of all brain voxels were included in VLSM (Fig. 4B). Bilateral anterior (especially left frontal) regions were not sufficiently covered largely due to the infrequent involvement of anterior circulation and exclusion of aphasic patients with lesions in the dominant hemisphere. Detailed statistics for all 14 VLSM analyses were listed in Table 3. Detailed results concerning number of significant voxels in each brain region were shown in Table 4. In the VLSM analysis for HDRS sum score, lesions in bilateral basal ganglia and bilateral capsular regions were found significantly associated with higher overall PSDS severity (Table 4, Fig. 4C). In the subsequent VLSM for individual PSDS, most of the above regions were also correlated with higher severities of *Depressed mood*, *Loss of interest* and *Psychiatric anxiety* (Table 4, Fig. 4D-F), but not with the severities of peripheral PSDS (Table 3). Peripheral symptoms could not be mapped to any certain brain region.

**Table 3:** Detailed statistics for all 14 VLSM analyses

Behavioral variable	Maximum Z value	FWE-corrected threshold	N of significant voxels	Location of significant voxels
HDRS sum score	5.216361	z > 4.32560	172	See Table 4, Fig. 4C
Individual PSDS score				
Depressed mood	5.178200	z > 4.46646	233	See Table 4, Fig. 4D
Feelings of guilt	4.511469	z > 4.47129	1	Unclassified^a^
Suicidality	7.111105	z > 6.86582	2	Unclassified^a^
Insomnia	3.995382	z > 4.52191	0	-
Loss of interest	5.321430	z > 4.52364	122	See Table 4, Fig. 4E
Retardation	4.828587	z > 4.96556	0	-
Agitation	4.526555	z > 4.85067	0	-
Psychiatric anxiety	5.295365	z > 4.42343	505	See Table 4, Fig. 4F
Somatic anxiety	4.656311	z > 4.94776	0	-
Gastrointestinal somatic	5.081490	z > 5.32943	0	-
General somatic	5.638429	z > 5.72118	0	-
Hypochondriasis	5.320743	z > 5.73211	0	-
Weight loss	5.980183	z > 5.77801	2	Unclassified^a^

*HDRS* indicates Hamilton Depression Rating Scale, *PSDS* post-stroke depression symptoms, *FWE* family-wise error. Positive Z when a lesion at the given voxel is correlated with a higher PSDS score

^a^unclassified indicates that the significant voxels do not belong to any grey matter region or white matter tract in AAL and “JHU-WhiteMatter-labels-1 mm” atlases

**Table 4:** Results of VLSM analyses for overall PSDS severity and individual PSDS severities

Variable	Associated brain region^a^	N of significant voxels
HDRS sum score	Left putamen	2
	Left pallidum	5
	Left posterior internal capsule	18
	Right putamen	27
	Right pallidum	66
	Right anterior internal capsule	11
	Right posterior internal capsule	18
	Right external capsule	17
	Right corona radiata	2
Individual PSDS score		
*Depressed mood*	Left pallidum	5
	Left posterior internal capsule	24
	Right putamen	10
	Right pallidum	90
	Right insula	7
	Right anterior internal capsule	40
	Right posterior internal capsule	24
*Loss of interest in work and activities*	Right pallidum	74
	Right anterior internal capsule	25
	Right posterior internal capsule	6
*Psychiatric anxiety*	Left pallidum	4
	Left posterior internal capsule	13
	Right caudate	4
	Right putamen	326
	Right pallidum	36
	Right insula	7
	Right anterior internal capsule	30
	Right external capsule	130
	Right corona radiata	55

*HDRS* indicates Hamilton Depression Rating Scale, *PSDS*, post-stroke depression symptoms

^a^brain regions with only one significant voxel were not reported

## Discussion

This study is among the first to visualize interactions among early-onset PSDS after stroke and unveil the strategic lesion locations for early-onset PSD at individual symptom level with a relatively large sample size.

In our relatively stable network, we identified three central PSDS at early stage of stroke. *Depressed mood* showed the highest centrality at early stage, which is in accordance with a recent study suggesting that *Depressed mood* remains the most central PSDS at discharge (20.19 ± 10.97 days after admission), 3 months and 12 months after discharge [10]. *Depressed mood* is also among the most central symptoms in neurologically healthy patients with major depressive disorder (MDD) [7, 8]. *Psychiatric anxiety* and *Loss of interest in work and activities*, however, are not included in the aforementioned study [10] which applied another depression rating scale (i.e. Center for Epidemiological Studies Depression Scale, CES-D), but may play key roles in PSDS network. Although we considered *Psychiatric anxiety* as a PSDS in this study, anxiety may also represent its own entity and correlate strongly with depression. Comorbidity between poststroke anxiety and PSD is common and well-established [27]. From the perspective of psychopathology, comorbidities of two mental disorders may arise due to shared symptoms between disorders [4]. These symptoms can act as causal bridges and influence symptoms of both PSD and poststroke anxiety at the same time [4]. *Psychiatric anxiety* is not included in DSM-5 criteria and CES-D, indicating that some diagnostic criteria or rating scales may not be able to cover all clinically relevant and central aspects of PSD [4]. The complex interactions between anxiety symptoms and PSDS in stroke patients are beyond the scope of our study and should be scrutinized in future studies. *Loss of interest* has also been reported as a central symptom in MDD patients [9]. Since central symptoms are considered more likely to be responsible for activating or maintaining the rest of symptoms, in-hospital psychosocial interventions targeting at the three central PSDS may achieve greatest benefits for stroke patients [4].

In our VLSM analysis in stroke patients, we found that lesions involving bilateral basal ganglia, bilateral posterior internal capsules, right anterior internal capsule and right external capsule were significantly associated with higher overall PSDS severity (Table 4). Patients with lesions involving posterior internal capsules commonly present with severe motor and somatosensory disturbances, and therefore high functional disability and psychosocial impact. Basal ganglia and related circuits play an important role in executive functions, behaviors and emotions [28]. The association between left basal ganglia strokes and PSD has been described in multiple studies [15]. Recent VLSM studies also reported right basal ganglia lesions to be associated with more PSDS [11, 29]. There is a growing consensus that strokes involving neural circuits connecting the prefrontal cortex, basal ganglia, thalamus, and amygdala (regardless of their lateralization) may disrupt executive function and mood regulation leading to PSDS [15], which is consistent with the frontal-limbic or prefrontal-subcortical theory described in MDD and vascular depression [30, 31]. Two important fiber pathways within the anterior internal capsules are disentangled: the anterior thalamic radiation (ATR), and the superolateral medial forebrain bundle (slMFB) [32]. Both pathways, as components of the prefrontal-subcortical system, play some roles in mood regulation [32]. Altered diffusion properties of the anterior internal capsule have been observed in MDD patients [33, 34]. Deep brain stimulation targeted on anterior internal capsule can effectively alleviate depressive symptoms among MDD patients [35]. The external capsule, containing cortico-cortical association fibers, has been reported to be disrupted in MDD patients [36, 37]. In the subsequent VLSM for individual PSDS, most of the above regions were also correlated with the severities of three central symptoms, but not peripheral PSDS (Table 4, Fig. 4). Peripheral symptoms are less likely to be associated with lesion location (Table 3).

Our results from network analysis and VLSM converge to a theory: basal ganglia and capsular strokes may first activate central symptoms and indirectly induce other PSDS via symptom-symptom interactions. The temporal order among PSDS cannot be determined with our cross-sectional behavioral data. Some researchers may argue that the most central node in a cross-sectional network may actually serve as a common endpoint of multiple causal chains and interventions targeting on the central symptom would have little effect on other nodes under this circumstance [38]. Assuming true in our case, however, it’s highly unlikely for the complex effect from multiple causal chains to “create” the relationship between lesion location and central PSDS when upstream nodes (i.e. peripheral symptoms) themselves are not associated with lesion location. Therefore, it’s more likely for the strategic lesion locations to first trigger central nodes and then indirectly involve the rest of nodes, resulting in higher overall PSDS severity. Although the stringent statistical method in VLSM may prove the role of strategic lesion locations in PSD, it should be noted that some psychosocial factors (e.g. disability) usually show higher associations with PSD than lesion location [2] and that the impact of psychosocial factors on the initiation and maintenance of PSDS network is yet to be elucidated.

Our findings conceptualize early-onset PSDS as a complex network model and extend the topic of neural substrates of PSD by combining network model with VLSM. Despite the cross-sectional nature of behavioral data in network analysis, the VLSM results can enhance the strength of causal inference from central to peripheral nodes. Our results may shed more light on the brain-behavior mechanisms and targeted interventions of PSD. Some limitations must also be noted. First, the HDRS may not cover all central aspects of PSD. For example, HDRS does not specifically assess *loss of energy*, which is a cardinal symptom of depression and may serve as another central node. Second, edge directionality cannot be determined and our group-level cross-sectional network may not be generalized to individual patients. The temporal order and directionality of interactions among PSDS have to be confirmed with longitudinal behavioral data [4]. Third, some brain regions (especially the left frontal region) are not sufficiently involved in our sample, leaving those regions unexplored or underpowered. Recent evidence suggests that about 3,000 stroke patients are required to achieve a lesion coverage of 86% of all brain voxels in VLSM [39]. Future studies with larger sample sizes or data sharing may overcome this limitation. Fourth, selection bias towards mild strokes and in particular to strokes without left hemispheric cortical involvement may limit the generalizability of our findings. Finally, the spatial topography information used in VLSM may represent a surface-level depiction of the lesion largely blind to its impact on the underlying brain networks [40]. Our work to unravel the network substrates of PSDS using lesion-network mapping [40, 41] or disconnectome [42] analysis is currently ongoing.

## Conclusions

*Depressed mood, Psychiatric anxiety and Loss of interest* are central PSDS at the early stage of stroke. The strategic lesion locations of central symptoms may indirectly induce other PSDS via symptom-symptom interactions, resulting in higher overall PSDS severity.

## Supplementary Information

**Additional file 1: Supplemental Table I. **Image Acquisition Protocols. **Supplemental Figure I. **Z-score standardized values of closeness, betweenness and strength for all nodes.Closeness centrality refers to the inverse sum of the lengths of the shortest paths from a node to all other nodes. Betweenness centrality is the number of times a node lies on the shortest path between two other nodes. Strength centrality is the sum of all absolute values of edges connected to a node. Betweenness and closeness have demonstrated poor reliability in psychopathology network studies. Expected influence considers both positive and negative edges and may be more reliable and interpretable than strength. DepMood indicates depressed mood; Guilt, guilt feelings; Suic, suicidality; Insomn, insomnia; Workact, loss of interest in work and activities; Retard, retardation; Agit, agitation; PsyAnx, psychiatric anxiety; SomAnx, somatic anxiety; GISom, gastrointestinal somatic symptoms; GenSom, general somatic symptoms; Hypochon, hypochondriasis; WtLoss, weight loss. **Supplemental Figure II.** Stability of betweenness, closeness and strength in 1,000 case-dropping bootstraps. CS-C for betweenness: 0.672; CS-C for closeness: 0.75; CS-C for strength: 0.75. **Supplemental Figure III. **Bootstrap edge weights difference test between non-zero estimated edge-weights in the network. Bootstrapped difference tests (α = 0.05) between edge-weights that were non-zero in the network. Significant differences between two edges are indicated by black boxes, non-significant differences are indicated by grey boxes. The color of the boxes (ranging from white to blue) corresponds to the thickness of the edge. DepMood indicates depressed mood; Guilt, guilt feelings; Suic, suicidality; Insomn, insomnia; Workact, loss of interest in work and activities; Retard, retardation; Agit, agitation; PsyAnx, psychiatric anxiety; SomAnx, somatic anxiety; GISom, gastrointestinal somatic symptoms; GenSom, general somatic symptoms; Hypochon, hypochondriasis; WtLoss, weight loss.

## Data Availability

The datasets generated and/or analysed during the current study are not publicly available due to ongoing data mining but are available from the corresponding author on reasonable request.

## Abbreviations

HDRS: Hamilton Depression Rating Scale
MDD: Major depressive disorder
PSD: Post-stroke depression
PSDS: Post-stroke depression symptoms
VLSM: Voxel-based lesion-symptom mapping
