# Supplementary material for: Psychopathological network for early-onset post-stroke depression symptoms
Source: BMC Psychiatry. 2023 Feb 21;23:114. doi: 10.1186/s12888-023-04606-1 (PMC9945689; doi:10.1186/s12888-023-04606-1)
Supplement: Supplementary file 1 — Additional file 1: Supplemental Table I. Image Acquisition Protocols. Supplemental Figure I. Z-score standardized values of closeness, betweenness and strength for all nodes.Closeness centrality refers to the inverse sum of the lengths of the shortest paths from a node to all other nodes. Betweenness centrality is the number of times a node lies on the shortest path between two other nodes. Strength centrality is the sum of all absolute values of edges connected to a node. Betweenness and closeness have demonstrated poor reliability in psychopathology network studies. Expected influence considers both positive and negative edges and may be more reliable and interpretable than strength. DepMood indicates depressed mood; Guilt, guilt feelings; Suic, suicidality; Insomn, insomnia; Workact, loss of interest in work and activities; Retard, retardation; Agit, agitation; PsyAnx, psychiatric anxiety; SomAnx, somatic anxiety; GISom, gastrointestinal somatic symptoms; GenSom, general somatic symptoms; Hypochon, hypochondriasis; WtLoss, weight loss. Supplemental Figure II. Stability of betweenness, closeness and strength in 1,000 case-dropping bootstraps. CS-C for betweenness: 0.672; CS-C for closeness: 0.75; CS-C for strength: 0.75. Supplemental Figure III. Bootstrap edge weights difference test between non-zero estimated edge-weights in the network. Bootstrapped difference tests (α = 0.05) between edge-weights that were non-zero in the network. Significant differences between two edges are indicated by black boxes, non-significant differences are indicated by grey boxes. The color of the boxes (ranging from white to blue) corresponds to the thickness of the edge. DepMood indicates depressed mood; Guilt, guilt feelings; Suic, suicidality; Insomn, insomnia; Workact, loss of interest in work and activities; Retard, retardation; Agit, agitation; PsyAnx, psychiatric anxiety; SomAnx, somatic anxiety; GISom, gastrointestinal somatic symptoms; GenSom, general somatic symptoms; Hypoc [file 12888_2023_4606_MOESM1_ESM.docx]

**SUPPLEMENTAL MATERIAL**

**Supplemental Table I. Image Acquisition Protocols**

|  | MRI | CT |
| --- | --- | --- |
| Tongji Hospital | GE’s Discovery MR750 3.0T scanner;  Axial plane;  T1WI: TR=2992.3ms, TE=24ms, TI=859ms, slice thickness =5mm, intersection gap =1.5mm, matrix =320*320, FOV =240*240mm^2^;  T2WI: TR=4650ms, TE=102ms, slice thickness =5mm, intersection gap =1.5mm, matrix =320*224, FOV =240*240mm^2^;  T2 FLAIR: TR=8000ms, TE=160ms, TI=2100ms, slice thickness =5mm, intersection gap =1.5mm, matrix =256*256, FOV =240*240mm^2^;  DWI: TR=3000ms, TE=65ms, slice thickness =5mm, intersection gap =1.5mm, matrix =256*256, FOV =240*240mm^2^; ADC map was derived from b0 and b1000 images. | GE’s Discovery CT750 HD; axial plane; The CT protocols were as follows: 120 kV, automatic tube current modulation (300 mAs), 5mm section interval, and 5mm section thickness; reconstruction section thickness =1.25mm, slices =112. |
| Wuhan Central Hospital | Siemens’ MAGNETOM Skyra 3.0T MRI scanner;  Axial plane;  T1WI: TR=2000ms, TE=11ms, TI=825ms, slice thickness =5mm, intersection gap =1.5mm, matrix =320*320, FOV =220*220mm^2^;  T2WI: TR=4000ms, TE=99ms, slice thickness =5mm, intersection gap =1.5mm, matrix =512*512, FOV =240*240mm^2^;  T2 FLAIR: TR=8000ms, TE=84ms, TI=2220ms, slice thickness =5mm, matrix =320*320, FOV =220*220mm^2^;  DWI: TR=4040ms, TE=64ms, slice thickness =5mm, intersection gap =1.5mm, matrix =160*160, FOV =220*220mm^2^; ADC map was derived from b0 and b1000 images. | UNITED IMAGING’s UCT760 scanner; axial plane, scan increment =5mm, slice thickness =5mm, rotation time =0.8s, tube voltage =120kV, tube current =285mA; reconstruction slice thickness =1.25mm, slices =125. |
| Wuhan First Hospital | Siemens’ MAGNETOM Vida 3.0T MRI scanner;  Axial plane;  T1WI: TR=1800ms, TE=11ms, TI=818ms, slice thickness =5mm, intersection gap =1.5mm, matrix =320*320, FOV =230*230mm^2^;  T2WI: TR=4500ms, TE=107ms, slice thickness =5mm, intersection gap =1.5mm, matrix =384*384, FOV =230*230mm^2^;  T2 FLAIR: TR=7000ms, TE=87ms, TI=2220ms, slice thickness =5mm, intersection gap =1.5mm, matrix =320*320, FOV =230*230mm^2^;  DWI: TR=4040ms, TE=64ms, slice thickness =5mm, intersection gap =1.5mm, matrix =160*160, FOV =230*230mm^2^; ADC map was derived from b0 and b1000 images. | Siemens’ SOMATOM Definition Flash CT scanner; axial plane, scan increment =5mm, slice thickness =5mm, rotation time =0.5s, tube voltage =120kV, automatic tube current modulation; reconstruction slice thickness =2.5mm, slices =48. |

T1WI indicates T1 weighted image; T2WI, T2 weighted image; T2 FLAIR, T2 fluid attenuated inverse recovery; DWI, diffusion weighted image; ADC, apparent diffusion coefficient; TR, repetition time; TE, echo time; TI, inversion time; FOV, field of view; MRI, magnetic resonance imaging; CT, computed tomography.


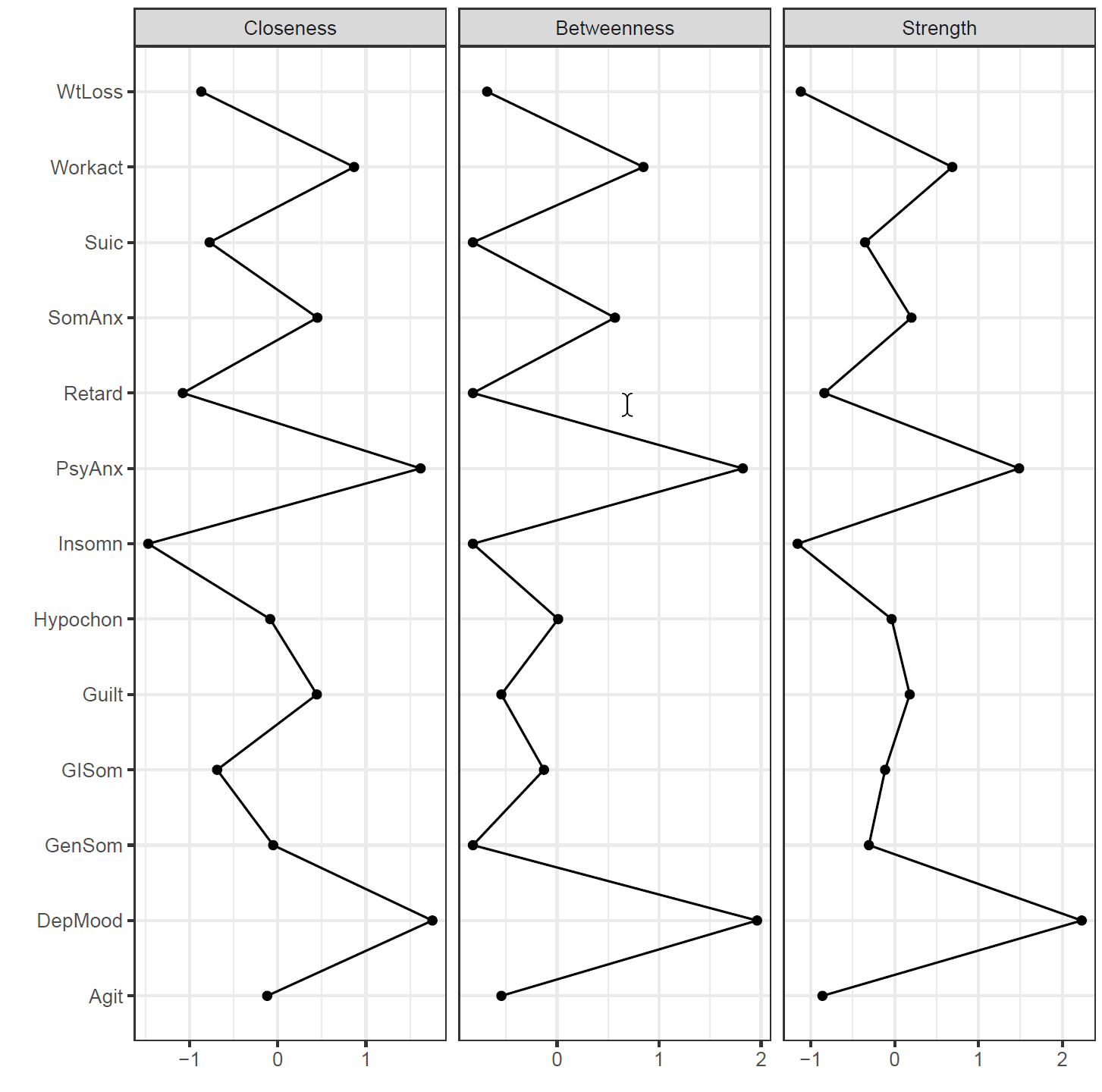


**Supplemental Figure I. Z-score standardized values of closeness, betweenness and strength for all nodes.** Closeness centrality refers to the inverse sum of the lengths of the shortest paths from a node to all other nodes. Betweenness centrality is the number of times a node lies on the shortest path between two other nodes. Strength centrality is the sum of all absolute values of edges connected to a node. Betweenness and closeness have demonstrated poor reliability in psychopathology network studies. Expected influence considers both positive and negative edges and may be more reliable and interpretable than strength.

DepMood indicates depressed mood; Guilt, guilt feelings; Suic, suicidality; Insomn, insomnia; Workact, loss of interest in work and activities; Retard, retardation; Agit, agitation; PsyAnx, psychiatric anxiety; SomAnx, somatic anxiety; GISom, gastrointestinal somatic symptoms; GenSom, general somatic symptoms; Hypochon, hypochondriasis; WtLoss, weight loss.


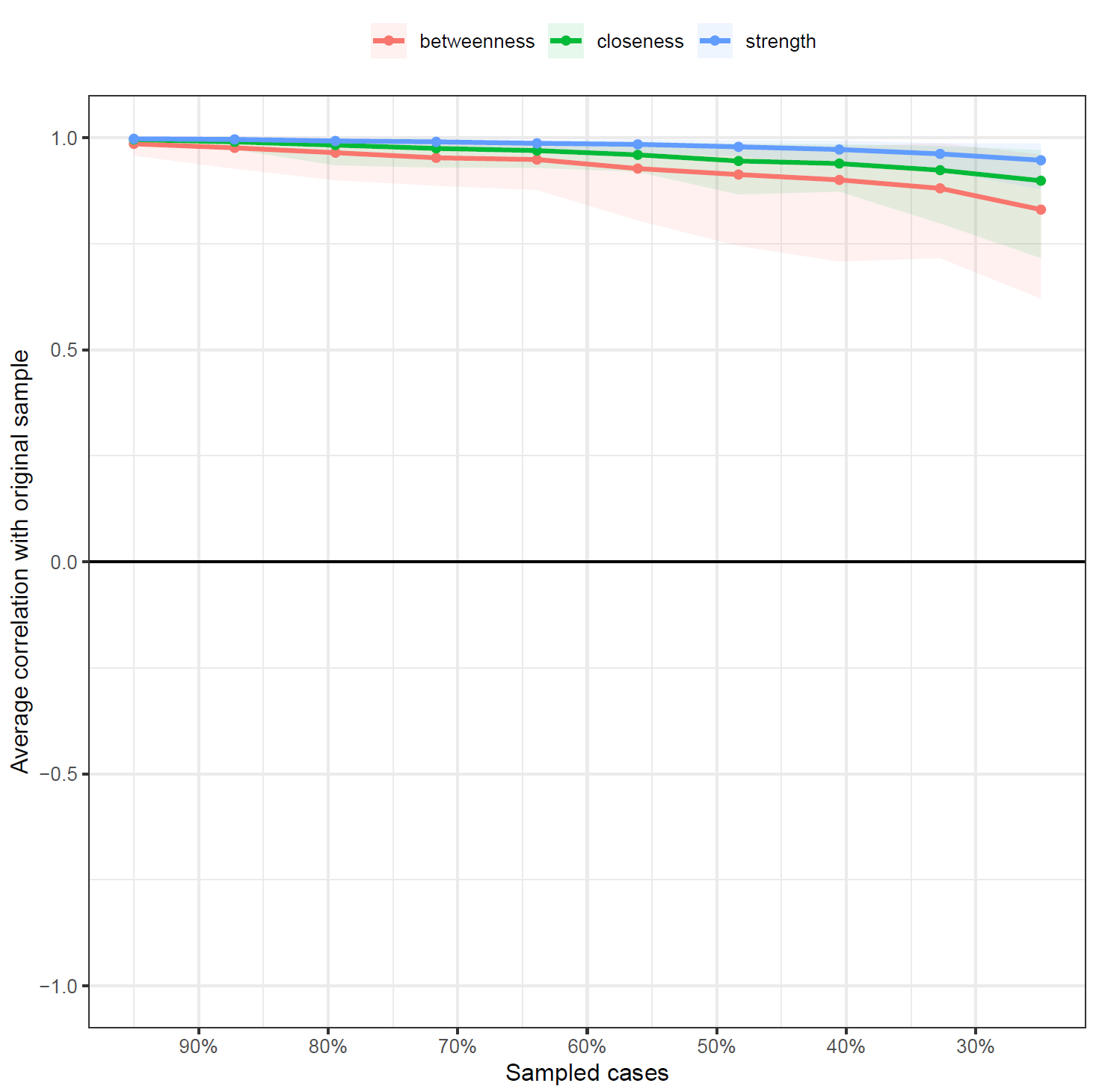


**Supplemental Figure II. Stability of betweenness, closeness and strength in 1,000 case-dropping bootstraps.** CS-C for betweenness: 0.672; CS-C for closeness: 0.75; CS-C for strength: 0.75.


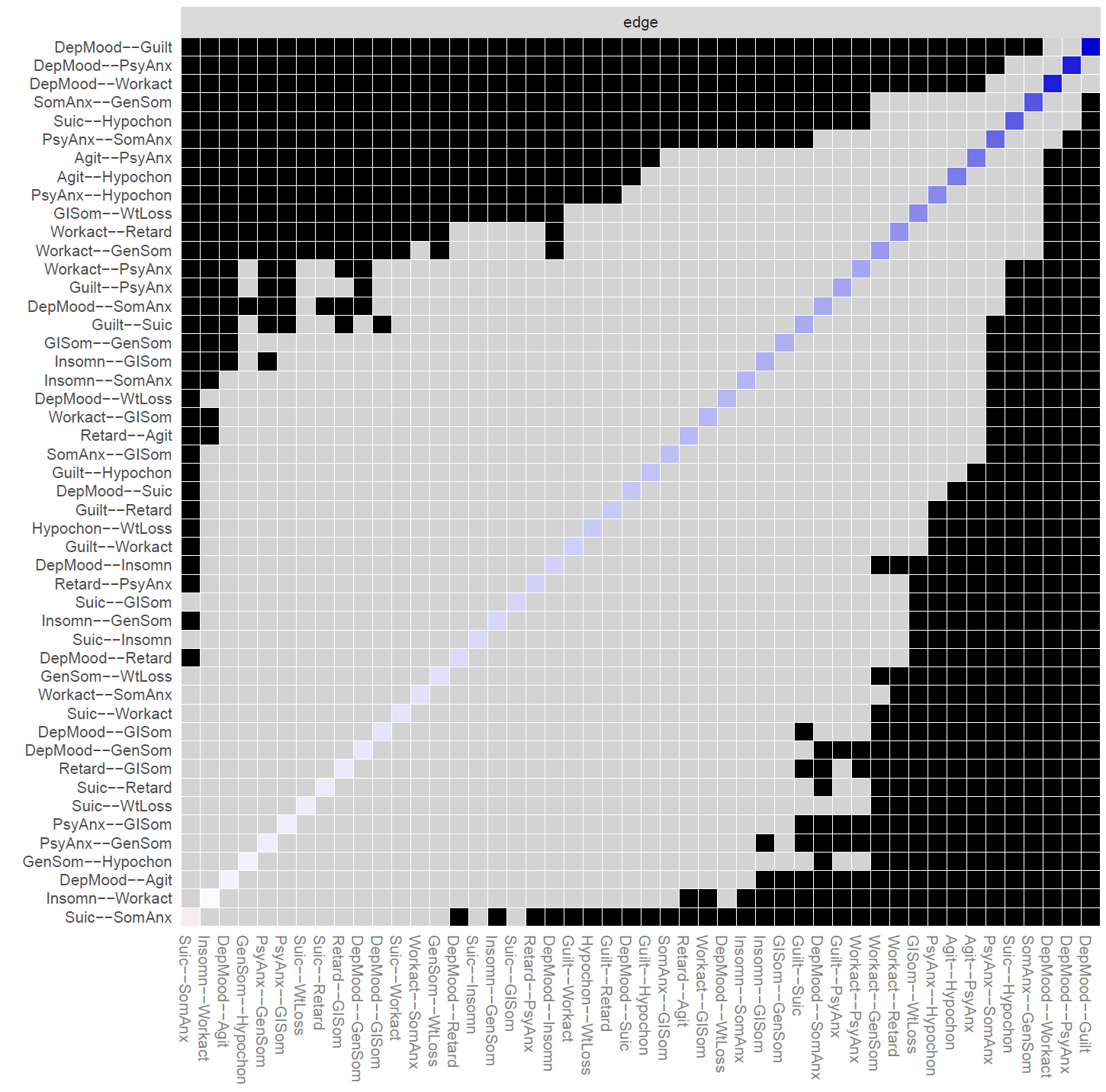


**Supplemental Figure III. Bootstrap edge weights difference test between non-zero estimated edge-weights in the network.** Bootstrapped difference tests (α = 0.05) between edge-weights that were non-zero in the network. Significant differences between two edges are indicated by black boxes, non-significant differences are indicated by grey boxes. The color of the boxes (ranging from white to blue) corresponds to the thickness of the edge.

DepMood indicates depressed mood; Guilt, guilt feelings; Suic, suicidality; Insomn, insomnia; Workact, loss of interest in work and activities; Retard, retardation; Agit, agitation; PsyAnx, psychiatric anxiety; SomAnx, somatic anxiety; GISom, gastrointestinal somatic symptoms; GenSom, general somatic symptoms; Hypochon, hypochondriasis; WtLoss, weight loss.
